# Taxonomic Identity Resolution of Highly Phylogenetically Related Strains and Selection of Phylogenetic Markers by Using Genome-Scale Methods: The *Bacillus pumilus* Group Case

**DOI:** 10.1371/journal.pone.0163098

**Published:** 2016-09-22

**Authors:** Martín Espariz, Federico A. Zuljan, Luis Esteban, Christian Magni

**Affiliations:** 1 Instituto de Biología Molecular de Rosario (IBR-CONICET), Suipacha 590, (S2002LRK) Rosario, Argentina; 2 Facultad de Ciencias Bioquímicas y Farmacéuticas, Universidad Nacional de Rosario, Suipacha 531, (S2002LRK) Rosario, Argentina; 3 Departamento de Fisiología, Facultad de Ciencias Médicas, Universidad Nacional de Rosario (UNR), Santa Fe 3100, (S2002LRK) Rosario, Argentina; Loyola University Chicago, UNITED STATES

## Abstract

*Bacillus pumilus* group strains have been studied due their agronomic, biotechnological or pharmaceutical potential. Classifying strains of this taxonomic group at species level is a challenging procedure since it is composed of seven species that share among them over 99.5% of 16S rRNA gene identity. In this study, first, a whole-genome *in silico* approach was used to accurately demarcate *B*. *pumilus* group strains, as a case of highly phylogenetically related taxa, at the species level. In order to achieve that and consequently to validate or correct taxonomic identities of genomes in public databases, an average nucleotide identity correlation, a core-based phylogenomic and a gene function repertory analyses were performed. Eventually, more than 50% such genomes were found to be misclassified. Hierarchical clustering of gene functional repertoires was also used to infer ecotypes among *B*. *pumilus* group species. Furthermore, for the first time the machine-learning algorithm Random Forest was used to rank genes in order of their importance for species classification. We found that *ybbP*, a gene involved in the synthesis of cyclic di-AMP, was the most important gene for accurately predicting species identity among *B*. *pumilus* group strains. Finally, principal component analysis was used to classify strains based on the distances between their *ybbP* genes. The methodologies described could be utilized more broadly to identify other highly phylogenetically related species in metagenomic or epidemiological assessments.

## Introduction

The highly phylogenetically related *B*. *pumilus* group is composed by *B*. *pumilus*, *B*. *safensis*, *B*. *altitudinis*, *B*. *stratosphericus*, *B*. *aerophilus*, *B*. *xiamenensis*, and *B*. *invictae* species that share more than 99% of its 16S rRNA gene sequence similarity. An increasing number of genome sequences from *B*. *pumilus* group strains are becoming available, since these bacteria have wide range of agronomic, biotechnological, and pharmaceutical uses [1–11]. However, strains of this group are frequently misnamed, precluding the possibility of performing predictive or comparative analysis [12].

Currently, whole genome sequences are obtained in a faster, cheaper, and more reliable way than was possible previously and can be accessed via public databases [13]. Concomitantly, bioinformatics tools were developed to use these data in an attempt to circumscribe bacterial species. These include the *in silico* DNA-DNA hybridization H (*is*-DDH), average nucleotide identity (ANI) among shared genes, tetranucleotide frequency correlation coefficients, and multilocus sequence analysis (MLSA) using the core genome of a genus [14,15]. The common characteristic of these genome-scale techniques is that they relay the confidence of the genome sequence assignment used as reference. Unfortunately, to upload a genome sequence, rigorous quality control regarding its taxonomic identity is not required. While there are well-curated genomic database [16], many genomes deposited in public databases are misnamed, mainly because of the common practice of identifying strains using 16S rRNA gene sequence data alone [17].

In this study, we used information available from databases to resolve the identity of *B*. *pumilus* group strains at a species level. In order to attempt this, we first determined the identity of available genome sequences using ANI correlation and core-based phylogenomic analyses. In addition, we performed a hierarchical cluster analysis based on gene function repertoires. Moreover, the Random Forest (RF) algorithm was used to rank genes based on their performance as phylogenetic markers, and principal component analysis (PCA) was conducted to accurately predict species identities by using genetic distances for the most important genes.

## Materials and Methods

### Nucleotide sequence data

All genomes used in this work are listed in Table 1 and S1 Table. For the construction of the pipelines, we included all available genome sequences from the *B*. *pumilus* group (accessed January 2015). For comparative proposes, genomic data from *B*. *amyloliquefaciens* subsp. *plantarum* FZB42(T) and *B*. *subtilis* 168(T) were also included.

**Table 1:** Proposed species names and assembly data for strains used for pipeline construction and testing.

Species	Strain^1^	Proposed new species name	Genome size (Mbp)	Number of contigs	Number of predicted CDSs	GenBank assembly accession	Random forest Group
***B*. *xiamenensis***	**HYC-10(T)**	*B*. *xiamenensis*	3.61	134	3,590	AMSH00000000.1	Train
*B*. *pumilus*	B4133	*B*. *altitudinis*	3.72	56	3,680	JXCN00000000.1	Test
*B*. *aerophilus*	C772		3.75	17	3,705	JXRO00000000.1	Train
*B*. *pumilus*	INR7		3.68	55	3,657	AYTK00000000.1	Test
*B*. *altitudinis*	B-388		3.71	59	3,598	JOVS00000000.1	Train
*B*. *stratosphericus*	LAMA 585		3.71	19	3,717	APAS00000000.1	Train
*B*. *pumilus*	MTCC B6033		3.76	1	3,680	GCA_000590455.1	Test
***B*. *altitudinis***	**41KF2b(T)**		3.68	39	3,672	ASJC00000000.1	Train
*B*. *pumilus*	BA06		3.75	15	3,688	AMDH00000000.1	Test
*B*. *pumilus*	S-1		3.69	144	3,697	AGBY00000000.1	Test
*B*. *pumilus*	B4129	*B*. *safensis*	3.67	23	3,675	JXCM00000000.1	Test
*B*. *safensis*	S9		3.79	22	3,900	LIHF01000000	Train
*B*. *pumilus*	WP8		3.71	1	3,607	GCA_000800825.1	Test
***B*. *safensis***	**FO-36b(T)**		3.73	37	3,719	ASJD00000000.1	Train
*B*. *pumilus*	B4134		3.68	19	3,638	JXCO00000000.1	Test
*B*. *pumilus*	B4107		3.65	29	3,610	JXCK00000000.1	Test
*B*. *pumilus*	CCMA-560		3.84	72	3,860	AUYP00000000.1	Test
*B*. *safensis*	VK		3.68	39	3,458	AUPF00000000.1	Train
*B*. *safensis*	CFA06		3.77	65	3,696	JNBO00000000.1	Train
*B*. *pumilus*	Fairview		3.83	39	3,744	JFBY00000000.1	Test
*B*. *pumilus*	7P	*B*. *pumilus*	3.57	8	3,455	JOJX00000000.2	Test
*B*. *pumilus*	SAFR-032		3.70	1	3,562	GCA_000017885.1	Train
***B*. *pumilus***	**ATCC 7061(T)**		3.83	16	3,730	ABRX00000000.1	Train
*B*. *pumilus*	B4127		3.89	46	3,885	JXCL00000000.1	Test
***B*. *amyloliquefaciens***	**FZB42(T)**		3.92	1	3,644	GCA_000015785.1	-
***B*. *subtilis***	**168(T)**		4.17	36	4,320	JNCM00000000.1	-

^1^ Type strains (T) are highlighted in bold

### ANI calculation and correlation analysis

ANI values were calculated as described by Repizo *et al*. [18] by using the JSpecies software with the BLAST algorithm [14]. The Pearson correlation matrix was conducted using the built in R package “stats” [19], and the correlation plot was constructed and ordered by hierarchical clustering using the R package “corrplot” [20].

### *In silico* DNA-DNA hybridization calculation

Estimates of *is-*DDH were made using the Genome BLAST Distance Phylogeny (GBDP) 2.0 Web server (http://ggdc.dsmz.de/distcalc2.php), and whole sequence length formulae *d*_0_ and *d*_6_ are described in Meier-Kolthoff *et al*. [15].

### Phylogenomic tree construction

Orthologous genes were assigned using all 4175 CDS from *B*. *subtilis* 168(T) as queries for bidirectional best-hit BLAST searches [21] against the CDS of all bacterial genomes under study (Table 1) and an *E-*value of 1E^-30^. Orthologous genes present in all microorganisms (BLAST defined core genes) were individually aligned using ClustalW2 [22], and concatenated using the Perl script catfasta2phyml.pl (http://www.abc.se/~nylander/catfasta2phyml/). The alignment was trimmed using GBlock 0.91b [23] and used to infer the evolutionary history of the strains with the Randomized Axelerated Maximum Likelihood algorithm (RAxML [24]), and the GTRGAMMAX model. This model was selected using jModelTest 2 software [25]. Reliability of the inferred tree was tested by bootstrapping with 1000 replicates.

### Hierarchical clustering and dendrogram comparison

Biological functions of proteins were inferred by correlation with orthologous group assignment using the OrthoMCL software [26] and an *E-*value of 1E^-5^. In the case that a particular species had more than one protein from the same group of orthologs, only the protein with the lower *E*-value was considered for the cluster analysis. In the case that OrthoMCL did not assign an orthologous group to a particular protein, its function was correlated from its best matching OrthoMCL-DB protein. Presence or absence of particular biological functions in the microorganisms were used as a binary scoring method (function present in a given strain = 1, absent = 0) and analyzed by average hierarchical clustering implemented using the R package “pvcluster” [27]. Distance measurements were calculated using the Manhattan distance function. Phylogenomic and functional dendrograms were compared and visualized with the R package “dendextend” [28].

### Training and evaluation of decision tree forests and determination of gene importance for bacteria classification

**Fig 1:**
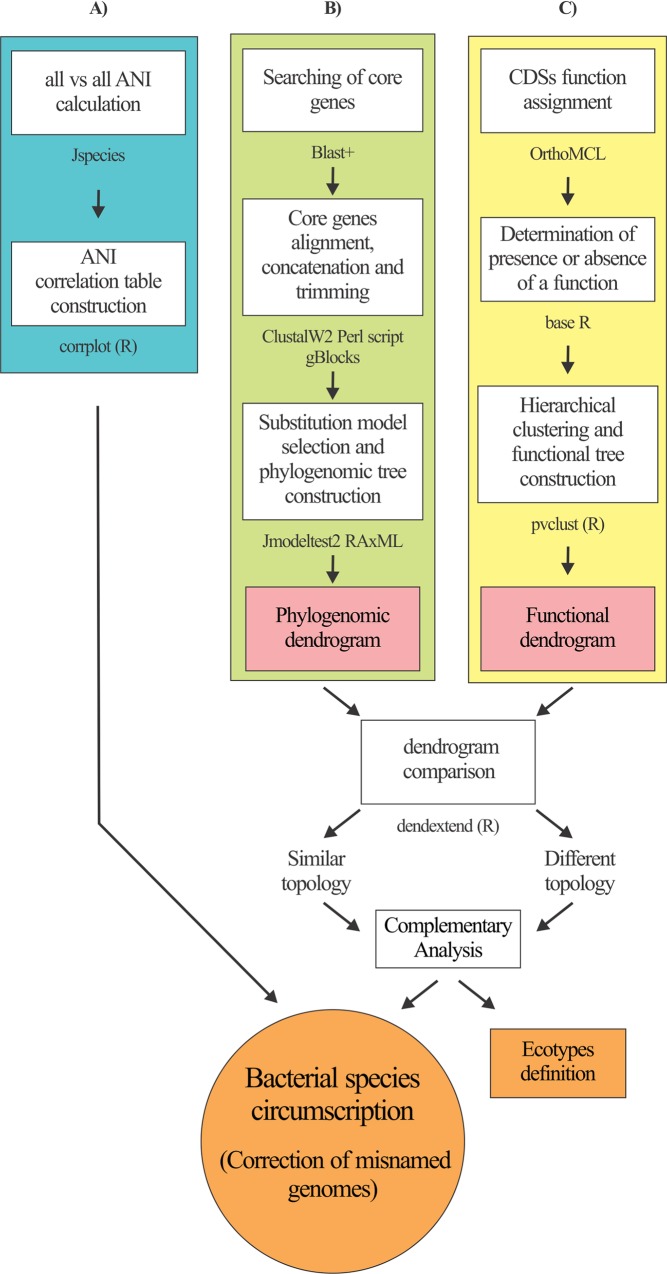
Pipeline for circumscription of *B*. *pumilus* group strains. The diagram describes the informatics tools used and the pipeline integrating genomic (A), phylogenomic (B) and functional (C) approaches for bacteria circumscription. A) *ANI approach*. ANI values of any two genomes among strains under study were calculated and then used to perform a correlation analysis. B) *Phylogenomic approach*. Core genes were searched using BLAST in all bacterial genomes under study. Orthologous genes were individually aligned, concatenated, and trimmed. Finally, the best substitution model was selected, and the evolutionary history inferred. C) *Encoded function repertoires approach*. The functions of all codified protein analyzed were assigned, and the presence or absence of particular biological functions in each of the microorganisms was determined. Finally, this binary information was used to perform a hierarchical cluster analysis. Similarities or differences between phylogenomic (B) and functional (C) dendrograms were used to define ecologically distinct strains, or reinforce a species definition. When necessary, complementary analyses like *is*-DDH were performed.

For the construction of decision tree forests, the RF algorithm was used. Distances between BLAST-defined core genes were calculated using the R package “ape” [29] and used as variables. The classes (or outputs) used were the suggested names of the species resulting from the genomic, phylogenomic and functional cluster analysis (following the pipeline described in Fig 1). Eleven strains were arbitrarily selected and used to train each forest (Table 1). For this, 100000 classification trees were constructed with a seed value of 12345. The importance of the variables was computed using internal out-of-bag estimates as described by Breiman [30]. The 13 strains from the *B*. *pumilus* group that had not been used to train the forests were used as a test set (Table 1) to construct a confusion table and calculate its misclassification rate.

### Clustering and outclass strain detection through PCA using genetic distances of most important genes

For the PCA, the genetic distances of *ybbP* of the strains under study (listed in Table 1 and S1 Table) were computed with the R package “ape” [29]. The PCA was conducted using the R built-in package “stats” [19], and distances were used as variables. Principal component 1 (PC1) vs. principal component 2 (PC2), and 95% confidence interval ellipses for each class were plotted with the R package “ggbiplot” [31].

## Results and Discussion

### Circumscription of *Bacillus pumilus* group strains in species using whole-genome data

To resolve the taxonomic identity of strains of the *Bacillus pumilus* group, a pipeline to circumscribe them at species level was employed. This pipeline integrated genomic, phylogenomic and functional information (Fig 1). First, ANI values of any two genomes among *B*. *pumilus* group strains were calculated. As the ANI cut-off value for bacterial species demarcation is not precisely established, we performed a correlation analysis to cluster related taxa (Fig 1A). We compared the information obtained using these analyses with the evolutionary history of the strains that was inferred using an MLSA analysis (Fig 1B). To reduce the effect of differences in evolution rates, or the presence of horizontally acquired genes, only core genes were used in the analysis. Additionally, with the aim of filtering out horizontally acquired genes post-speciation, we also defined the gene core from strains of *B*. *subtilis* 168(T) and *B*. *amyloliquefaciens* FZB42(T). Finally, we performed hierarchical cluster analysis based on the codified function repertoires of microorganisms (Fig 1C). It should be highlighted that assignment of proteins or genes to given orthologous groups is a critical but challenging procedure [32]. For this assignment, we used the OrthoMCL algorithm that has been shown to accurately predict protein function, and the OrthoMCL-DB database that contains 1398546 proteins and 150 genomes including eukaryotes, archaea and prokaryotes ([33], http://www.orthomcl.org). In this pipeline, coherence between MLSA and functional dendrograms was used to reinforce a given bacterial species circumscription. Different topologies were used to recognize ecologically distinct strains of a given monophyletic group (Fig 1).

#### The ANI approach

**Fig 2:**
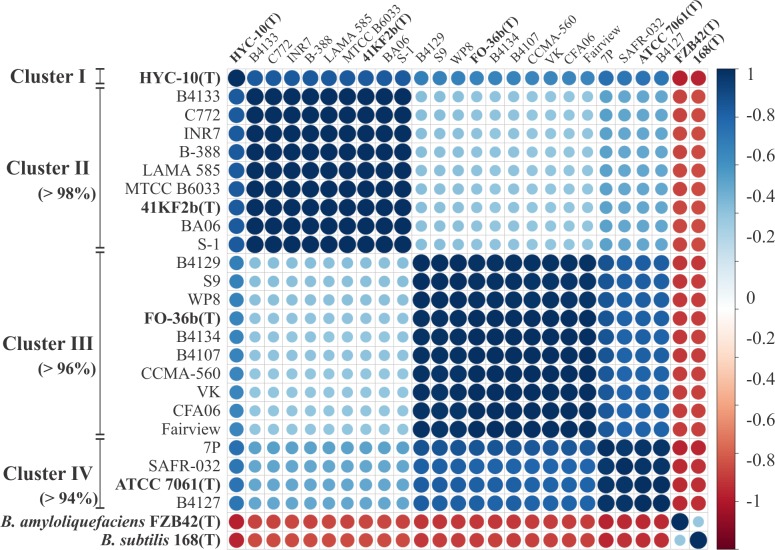
Correlation plot based on strain ANI values. ANI values between each indicated strain (Types in bold) were calculated using the JSpecies software [14] and used for a Pearson correlation matrix construction conducted using R [19]. The plot shows the correlation constructed and ordered by hierarchical clustering using the R package “corrplot” [20]. The minimum percentages of ANI values between strains of a given cluster are indicated in brackets.

The ANI analysis performed as described in Materials and methods, and depicted in Fig 2 shows that *B*. *pumilus* group strains cluster in four different sub-groups. One of these groups is composed of a single strain, *B*. *xiamenensis* HYC-10(T), which shared less than 91% ANI with the other strains (S2 Table). We also observed that two strains of *B*. *altitudinis* (the type strain 41KF2b(T) and B-388) cluster together with five strains assigned as *B*. *pumilus* (B4133, INR7, MTCC B6033, BA06 and S-1), *B*. *aerophilus* (C772) and *B*. *stratosphericus* (LAMA 585) (Fig 2). These strains shared more than 98% ANI with each other and less than 90% with members of other clusters (S2 Table). A third cluster was also found composed of six and three strains assigned as *B*. *pumilus* (B4129, WP8, B4134, B4107, and CCMA-560) and *B*. *safensis* (the type strain FO-36b(T), S9, and VK), respectively (Fig 2). All cluster III members shared more than 96% ANI and less than 93% with other clusters. The last cluster consisted of four strains from the *B*. *pumilus* species (the type strain ATCC 7061(T), 7P, SAFR-032, and B4127) (Fig 2). However, the ANI values shared between these strains were close to the ±94% considered as the ANI boundary for the taxonomic circumscription of prokaryotic species [34] (S2 Table). Hence, *is*-DDH values were computed to evaluate whether cluster IV circumscribes strains from the same species. For this, DDH were predicted using the GBDP web tool and whole genome formulae, obtaining values of over 87%, which were larger than the 70% generally assumed to be the cut-off for species demarcation (S3 Table) [15].

#### The phylogenomic approach

**Fig 3:**
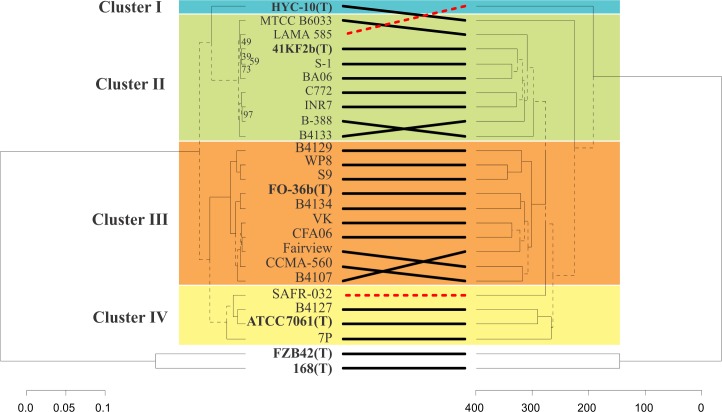
Comparison of phylogenomic and functional dendrograms of *Bacillus pumilus* group strains. Phylogenomic and functional dendrogram comparisons were performed and plotted with the R package “dendextend” [28]. A) *Phylogenomic dendrogram*. 109 BLAST core genes were individually aligned, concatenated and trimmed resulting in a final alignment containing a total of 104022 residues. The evolutionary history of the indicated strains was inferred with RAxML algorithm [24]. Reliability of the inferred tree was tested by bootstrapping with 1000 replicates. When not indicated, the bootstrap support values were 100. B) *Functional dendrogram*. Biological functions of proteins encoded in the genome of the indicated strain (Types in bold) were inferred using the OrthoMCL software [26] and then used as a binary score for hierarchical cluster analysis implemented with the R package “pvcluster” [27].

One hundred and nine conserved genes (listed in S4 Table) were found with a reciprocal best-hit BLAST search, using all CDS of *B*. *subtilis* 168(T) as a search query. Our approach was based on the assumption that these genes belonged to the *Bacillus* genera core, were not transferred horizontally post-speciation, and have evolved concomitantly following a similar topology to the species under analysis. However, the existence of such non-transferable genes or even the concept of core genes is under discussion [35]. Nevertheless, the number of core genes found were very similar to the current 44 putative core genes identified for Eubacteria [35]. From a reliability perspective, this is more than the 20 genes proposed to be sufficient to provide high-confidence phylogenetic reconstruction [36]. Remarkably, the phylogenomic tree constructed on the basis of the alignment of these 109 core genes showed similar clustering to *B*. *pumilus* group strains obtained based on ANI values (Fig 3, left tree).

#### The functional repertoire approach

**Fig 4:**
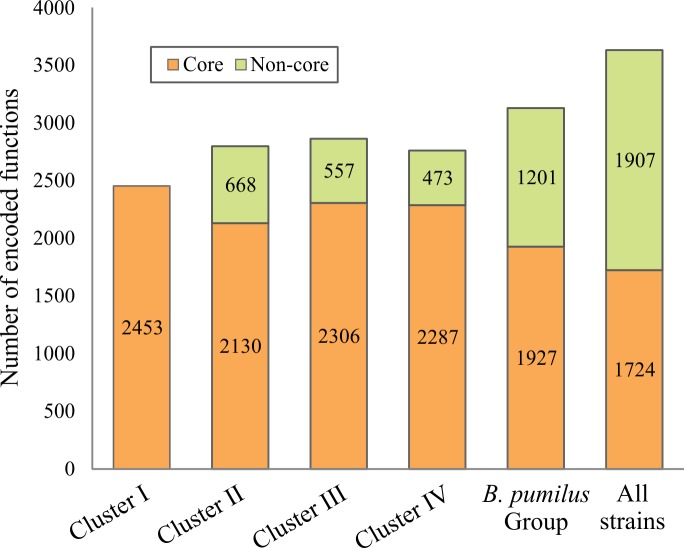
Analysis of functional repertoires among clusters of *Bacillus pumilus* group strains. Numbers of biological functions of proteins encoded in each cluster, all *B*. *pumilus* group strains or all 26 strains under analysis are indicated.

An ecotype is defined as a genetically cohesive group that shares genetic adaptations to a particular set of habitats, resources, and conditions [17,37]. The ecotype concept has been recently proposed as rational basis for demarcating bacterial taxa [17,34,38]. As different ecotypes could be identified by comparing genome content [17] the function of all CDS of strains under analysis (Table 1) were assigned and compared. As a result, 3128 different functions were identified for the 88223 CDS analyzed, 2731 of which were associated with an OrthoMCL-DB orthologous group and the 397 remaining were assigned on the basis of the best hit to OrthoMCL-DB proteins without a defined orthologous group (Fig 4). Amongst the *B*. *pumilus* group strains, 1927 functions were found that represent the core functions of the phylogenetic group. When *B*. *subtilis* 168(T) and *B*. *amyloliquefaciens* FZB42(T) were included in the analysis, 1724 common functions were found (Fig 4). This value was significantly higher than the 109 core genes found using a BLAST reciprocal best-hit search. This discrepancy highlights the dissimilar criteria used by both methodologies (see Materials and methods). Moreover, the hierarchical cluster analysis constructed based on the functional repertoires among the 26 strains were mainly determined by the 1907 non-core functions, rather than by the common functions. Remarkably, in Fig 3 it is shown that both cluster analyses resulted in very similar topologies. Interestingly, strains SAFR-032 and LAMA 585 did not cluster with Cluster II and IV, respectively (right tree in Fig 3). SAFR-032 seemed to be close to the rest of Cluster IV strains, and a closer association could not be observed because of this cluster being less conserved, as the ANI and phylogenetic approach suggested. On the other hand, LAMA 585 was relatively distant from Cluster IV. This discrepancy is the result of the loss of 171 functions that all Cluster II strains except LAMA 585 have, and the acquisition or conservation of 28 functions that were found only in the latter. Our hypothesis is that these functions could have been lost or gained because of genome deletions or horizontal gene transfer events that allow the bacterium to adapt to a specific environment.

#### Integration of genomic, phylogenomic and functional approaches

Inconsistencies between current species assignment and the three clustering approaches described above suggested that at least 13 out of the 24 *B*. *pumilus* group strains are currently misnamed in databases. Cluster I is composed of a single Type strain of the species *B*. *xiamenensis* and therefore there are no arguments to invalidate its assignment. Conversely, according to database information, Cluster II was integrated by at least four different species. We suggest that strain members of this cluster should be assigned as *B*. *altitudinis* since group together with the Type strain 41KF2b(T). Concordantly, recently BA06 and S-1 strains were associated and proposed to be *B*. *altitudinis* species [12]. *B*. *invictae* Bi.FFUP1T, a member of the *B*. *pumilus* group was also renamed, based on its *is*-DDH and ANI values, and phenotypic analysis, as a *B*. *altitudinis* strain [39]. These facts clearly indicate that this taxonomic rank will continue to evolve. Regarding LAMA 585, we suggest that this strain could be classified as a different ecotype of the *B*. *altitudinis* species. To further support this, *is*-DDH between LAMA 585 and other members of Cluster II were calculated using GGDC 2.0 and whole sequence length formulae [15]. Values of over to 91% were obtained for LAMA 585 and any Cluster II member (S3 Table) that supports our hypothesis. Moreover, Branquinho *et al*. have recently proposed that the species *B*. *aerophilus* and *B*. *stratosphericus* should be rejected [40].

As our three independent approaches consistently indicated that all *B*. *safensis* strains, including the Type strain FO-36b(T) [41], were only found in Cluster III, we propose that the *B*. *pumilus* strains WP8 [5], B4134, B4107, CCMA-560 [42] and Fairview [43] should be renamed as *B*. *safensis*. Interestingly, while CCMA-560 is still designated as *B*. *pumilus* in GenBank and RefSeq databases, during the preparation of this manuscript, it was named as member of the *B*. *safensis* species in a recent publication [10].

Cluster IV are composed of *B*. *pumilus* species, including the Type strain ATCC 7061, and as discussed above they appear belong to the same species. However, ANI values among these strains (S2 Table) were close to the ±94% considered as the taxonomic boundary [34], and functional cluster analysis data was not consistent with a high degree of conservation among them. Therefore, the incorporation of new genome sequences may be needed to better describe the relationships between members of this cluster in terms of ecotypes, subspecies, or genomovars. Table 1 summarizes the new species assignment proposed based on our analysis.

### Phylogenetic marker selection using genetic distance data

**Fig 5:**
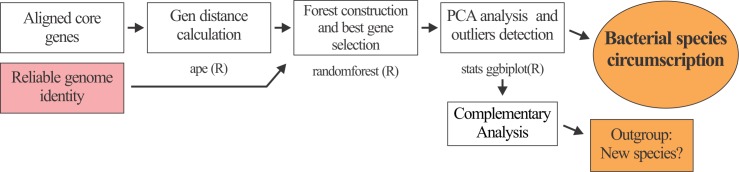
Pipeline to circumscribe bacteria as well as to rank genes base on their importance. First, gen distances among all individually aligned core genes are calculated. Then, a forest of decision trees is constructed considering all variables and as classes the suggested species names that resulted from the genomic, phylogenomic and functional cluster analysis (pipeline described in Fig 1). The importance of the variables are computed using RF algorithm [30]. Finally, distances of the most important gene are used to perform a PCA to circumscribe bacteria and identify outclasses. Further analysis (base on phylogenomic, genomic, and experimental phenotypic information) have to be performed to classify those outlier strains.

Selection of alternative marker genes that provide prokaryotic species boundaries at higher resolution than 16S rRNA is a challenging but necessary task to reconstruct genealogies [44,45]. Therefore, we analyzed the 109 *B*. *pumilus* group core genes defined in the phylogenomic approach described above to rank them and select the best-performing markers to circumscribe species in this taxonomic group. For this purpose, we used the RF algorithm as it is delineated in Fig 5. RF is a machine-learning algorithm that generates unpruned decision trees using a random subset of input variables. To classify a new object, each tree uses its input data to made a prediction (or “vote”), and the forest chooses the classification with the most votes [30]. In this study, we built a species classifier using RF and genetic distance as input data. We chose this algorithm since not only could it be used to measure variable importance, but it also runs fast and efficiently on large databases and does not require much fine-tuning of parameters, so the methodology is easily accessible for many users [30].

**Fig 6:**
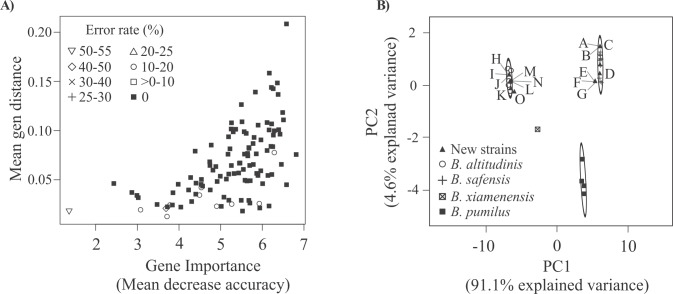
Ranking of genes based on their RF importance and PCA for outlier detection. A) *Importance and error rate plot*. Importance of each gene was computed using RF and plotted versus its gen distance mean. Symbols representing the percentage of the classification error rate are depicted. B) *PCA plot*. The PCA was conducted using R [19] and as variables, the distances to each of the *ybbP* orthologs from strains listed in Tables 1 and S4. PC1 vs. PC2 and 95% confidence interval ellipses were plotted with the R package “ggbiplot” [31]. Symbols used for strains listed in Table 1 are depicted in the figure. The 15 *B*. *pumilus* group strains listed in S4 Table (New strains) are depicted as closed triangles. A, JPL_MERTA2; B, RIT372; C, SCAL1; D, 15.1; E, LK12; F, LK21; G, LK32; H, LK31; I, LK18; J, W3; K, RIT380; L, LK23; M, LK33; N, LK5; O, DSM 26896.

To create the variables to construct the forest, we first calculated genetic distances between conserved genes (G) and their homologs in all strains or microorganisms (O) under study (Fig 5). This procedure generated a number of variables (V) equal to the total number of genes under analysis (or G x O). As we used the 109 BLAST-defined core genes of *B*. *pumilus* group as input data, the number of variables defined was 2834 (or 109 G x 26 O). Distances between orthologs were calculated using the R package “ape” [29] and then used to compute their means, variances, and maximums. While it was proposed that RF avoids overfitting [30], more recently it was indicated that special attention is required for some data distributions such as those with small sample sizes [46]. Therefore, we decided to use a subset of our input data to train the forest, and another independent subset to test its error rate. Microorganisms included in each subset are indicated in Table 1. The forest of decision trees was finally constructed considering all 2834 V, and as a response to the proposed species designation for the 11 strains of the training dataset. To evaluate the forest performance, the species of the 13 strains of the test dataset was predicted with the RF classifier and used to calculate the misclassification rate. We found that the identities of the strains were predicted accurately in all cases tested. The importance of each gene was obtained by using the internal out-of-bag estimation of the RF algorithm [30]. In Table 2, the 10 most important genes are indicated, and in S4 Table a complete ranked list of all 109 core genes of the *B*. *pumilus* group are indicated. Interestingly, ribosomal protein sequences (RPS) that were proposed to be used for resolving the whole bacterial domain at subspecies level [47] had the lowest importance index for the classification of *B*. *pumilus* group species. Moreover, RPS genes are the most conserved sequences, as indicated by the means of their genetic distances (S4 Table). In Fig 6A it is shown that there is a positive correlation between the mean of the genetic distance and the importance of the gene for the classification. Genes with a more recent evolutionary history that were implied in regulation, transport or sporulation functions in Firmicutes were more important for the classification of *B*. *pumilus* group strains. For example, the most important gene listed in Table 2, *ybbP* (more recently called *cdaA*) is conserved in nearly all Firmicutes (but not all bacteria), and seems to be responsible for the synthesis of the cyclic di-AMP, an essential secondary messenger that is required for cell wall homeostasis [48].

**Table 2:** Statistics of the 10 most important genes for RF species circumscription.

Genes	Gen distance mean^a^	Gen distance variance^a^	Gen distance maximum^a^	Importance^b^	Error rate^c^	Function^d^	Locus name^c^
*ybbP*	0.144	5.411E^-06^	0.668	6.813	0.000	CdaA diadenylate cyclase (c-di-AMP synthetase)	BSU01750
*ymdA*	0.084	6.996E^-07^	0.272	6.582	0.000	Ribonuclease Y	BSU16960
*yqjD*	0.137	2.778E^-06^	0.580	6.580	0.000	Propionyl-CoA carboxylase beta chain	BSU23920
*trmU*	0.148	8.676E^-06^	0.814	6.508	0.000	tRNA 2-thiouridylase	BSU27500
*yusV*	0.046	6.259E^-08^	0.209	6.489	7.692E^-02^	Iron(III)- siderophore transporter ATP binding component	BSU32940
*sigA*	0.100	5.368E^-07^	0.337	6.458	0.000	RNA polymerase sigma factor RpoD	BSU25200
*clpC*	0.054	2.023E^-07^	0.295	6.426	7.692E^-02^	Negative regulator of genetic competence ClpC/MecB	BSU00860
*spoIVA*	0.076	1.760E^-07^	0.281	6.393	0.000	Stage IV sporulation protein A	BSU22800
*yqhT*	0.057	7.763E^-08^	0.255	6.380	0.000	Peptidase YqhT	BSU24460
*tilS*	0.056	4.363E^-08^	0.204	6.361	0.000	tRNA(ile)-lysidine synthase	BSU00670

**a.** Distances of orthologs were calculated using the R package “ape” [29] and then used to compute their means, variances and maxima.

**b.** Importance of each gene was computed using internal out-of-bag estimates as described by Breiman [30] with a forest composed by 100000 classification trees trained by the 11 strains mentioned in Table 1, and the input data from all 109 core genes. The 10 most important genes are listed.

**c.** For error rate calculation, a new forest of 100000 classification trees was constructed for each gene and trained by the same dataset, but with distance data of the individual gene. Species identity of the 13 strains that belong to the test set (Table 1) were predicted and used to calculate the misclassification rate of each gene.

**d.** Function and locus names for each gene were obtained for the reference sequence NC_000964.3 of *B*. *subtilis* 168(T) [49].

As an attempt to evaluate the performance of each of the 109 core genes, their classification error rates were calculated. For this, 109 new forests of classification trees were constructed, one for each of the *B*. *pumilus* core genes. Each individual forest was trained by the distance data of the specific gene under analysis using the training dataset. Then, the species of the 13 strains in the test dataset were predicted using each individual classifier. The misclassification rate for each gene was estimated by comparing them with their true identities. As depicted in Fig 6A, more important, but also less conserved genes classified the strains of the test set most accurately.

To resolve the identity of poorly represented species (as *B*. *xiamenensis*) or even discover species that are not represented at all in the classifier, we decided to include a PCA in our pipeline (Fig 5). Therefore, a PCA was performed using *ybbP* distances to each of the 26 strains under analysis generating 26 variables. We found that strains of the same species were clustered together with 95% confidence, and that *B*. *xiamenensis*, *B*. *subtilis* 168(T), and *B*. *amyloliquefaciens* FZB42(T) were clearly identified as outliers (S1 Fig).

Finally, to exemplify how the pipeline described in Fig 5 works for bacteria circumscription we used information from 15 *B*. *pumilus* group genomes that became available during the preparation of this manuscript (last accessed June 2015, S4 Table). A PCA was run including the genetic distance values of the *ybbP* gene of the new strains. Fig 6B shows the plot of PC1 and PC2 that resulted from this analysis, and this explained the 91.1% and 4.6% variance, respectively. We found that strains LK31, LK18, LK23, LK33, LK5, W3, and RIT380 clustered with *B*. *altitudinis* strains and consequently should be assigned as a member of this species. On the other hand, strains JPL_MERTA2, RIT372, SCAL1, and 15.1 grouped with the *B*. *safensis* species (Fig 6B). Thus, our analysis suggested that strains SCAL1 and 15.1 should be renamed as *B*. *safensis*. It is worth mentioning that ANI, *is-*DDH and MLSA analyses for strains SCAL1, 15.1, LK31, LK18, W3, LK23, LK33, LK5, and RIT380 were consistent with our suggested species assignations (S5 Table and S2 Fig).

In Fig 6B it could also observed that strains LK12, LK21, LK32 and DSM 26896 were located outside of the 95% confidence ellipses defined by species with validated identity. Interestingly, DSM 26896 was the only bacterium of the *B*. *invictae* species with its genome sequence available. Hence, PCA was able to detect non-represented species that could be overlooked using RF. As strains LK12, LK21, and LK32 were named as *B*. *pumilus* but did not group together with strains of Cluster IV, they may belong to a different species. Noteworthy, ANI, *is-*DDH and MLSA analyses for LK12, LK21, and LK32 suggested that they are *B*. *safensis* strains (S5 Table and S2 Fig). The discrepancy with the PCA was due by the significance level used in the analysis. Nevertheless, it is important to note that to assign a new species identity to these strains, a comparative polyphasic analysis with reference strains that include phenotypic, genotypic, and phylogenetic approaches should be performed.

## Conclusions

The performance of new technologies in DNA sequencing as well as their low cost has resulted in a large number of genome sequences becoming available in a short time. However, the evolution of fast, standardized, and accurate procedures to properly identify such sequences at species level has yet to be established. This challenging task is crucial since only accurate classified genome data would guaranty reliable analysis in data mining or comparative genomics. Furthermore, misnaming the source of the available sequences would generate distortion in species description. For example, if a strain was isolated from an infection event and was incorrectly identified as being a member of a particular species, it could lead to the entire species being reported as potentially unsafe. This was the circumstance in which the safety of *B*. *pumilus* species had to be reviewed by European Food Safety Authority, owing to two instances of severe sepsis in neonatal infants caused by what was presumed to be a *B*. *pumilus* strain [50].

In this study, we first proposed 13 reassignments to the *B*. *pumilus* group strains used as references. We also suggested that the existence of non-identical topologies in phylogenomic and encoded function repertoire dendrograms might contribute to the definition of ecotypes. Finally, we made use of genetic distances and RF algorithms to rank and select gene markers for the construction of a species classifier based on a PCA. This procedure could be more broadly used for the accurate and reliable identification of highly related species. Moreover, selection of specific markers could be essential when no whole-genome information is available, such as in metagenomic studies where entire genomes could not be reconstructed, or during diagnostic or screening tests in epidemiological studies where a high number of samples need to be handled.

## Supporting Information

S1 Fig Species clustered by PCA. The PCA was conducted using the R package “stats” [19] and the distances to each of the *ybbP* orthologs from strains listed in Table 1 were used as variables. PC1 vs. PC2 and 95% confidence interval ellipses were plotted with the R package “ggbiplot” [31]. Symbols used for the strains listed in Table 1 are depicted.
(TIF)

S2 Fig Phylogenomic dendrogram of *Bacillus pumilus* group strains analyzed by PCA. 184 orthologous genes present in all microorganisms under analysis were individually aligned, concatenated and trimmed resulting in a final alignment containing a total of 153853 residues. The evolutionary history of the indicated strains was inferred with RAxML algorithm [24]. Reliability of the inferred tree was tested by bootstrapping with 1000 replicates. Type strains are indicated in bold.
(TIF)

S1 Table Proposed species names and assembly data for Strains used in PCA analysis. (XLSX)

S2 Table Percentage of ANI value between any two strains. ANI values were calculated as described by Repizo *et al*. [18] using the JSpecies software with BLAST algorithm [14]. Pearson correlation matrix was conducted using the R package “stats” [19], and the correlation plot was constructed and ordered by hierarchical clustering using the R package “corrplot” [20].
(XLSX)

S3 Table *is*-DDH analysis of LAMA 585 and Group IV strains. Estimates of is-DDH were made using the GBDP 2.0 Web server (http://ggdc.dsmz.de/distcalc2.php) and whole sequence length formulas *d*_*0*_ and *d*_*6*_ described in Meier-Kolthoff *et al*. [15].
(XLSX)

S4 Table Statistics for the 109 core genes. **a.** Distances of orthologs were calculated using the R package “ape” [29] and used to calculate their means, variances and maxima. **b.** Importance of each gene was calculated using internal out-of-bag estimates as described by Breimen [30] with a forest composed by 100000 classification trees trained by the 11 strains mentioned in Table 1, and the input data of all 109 core genes. **c.** To calculate the rate of errors, a new forest of 100000 classification trees was constructed for each gene and trained by the same dataset, but with distance data for the specific gene. The species of the 13 strains mentioned as test set in Table 1 were predicted and used to calculate the misclassification rates of each gene. **d.** Function and locus names for each gene were obtained for the reference sequence NC_000964.3 of *B*. *subtilis* 168(T) [49].
(XLSX)

S5 Table Percentage of ANI and *is*-DDH values between strains with proposed new species assignations and type strains. ANI values were calculated as described by Repizo *et al*. [18] using the JSpecies software with BLAST algorithm [14]. *is*-DDH were estimated using the GBDP 2.0 Web server (http://ggdc.dsmz.de/distcalc2.php) and *d*_*6*_ formulae [15].
(XLS)
