# Supplementary figures and images for: Taxonomic Identity Resolution of Highly Phylogenetically Related Strains and Selection of Phylogenetic Markers by Using Genome-Scale Methods: The Bacillus pumilus Group Case
Source: PLoS One. 2016 Sep 22;11(9):e0163098. doi: 10.1371/journal.pone.0163098 (PMC5033322; doi:10.1371/journal.pone.0163098)

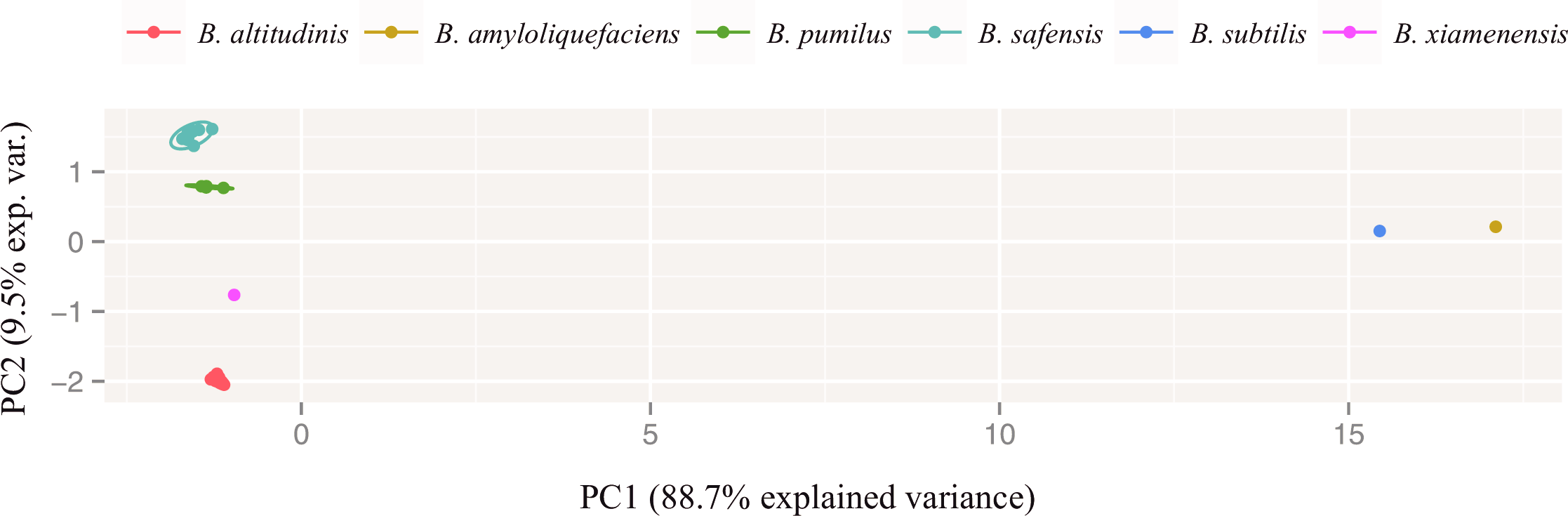

Supplement: S1 Fig — The PCA was conducted using the R package “stats” [19] and the distances to each of the ybbP orthologs from strains listed in Table 1 were used as variables. PC1 vs. PC2 and 95% confidence interval ellipses were plotted with the R package “ggbiplot” [31]. Symbols used for the strains listed in Table 1 are depicted. (TIF) [file pone.0163098.s001.tif]

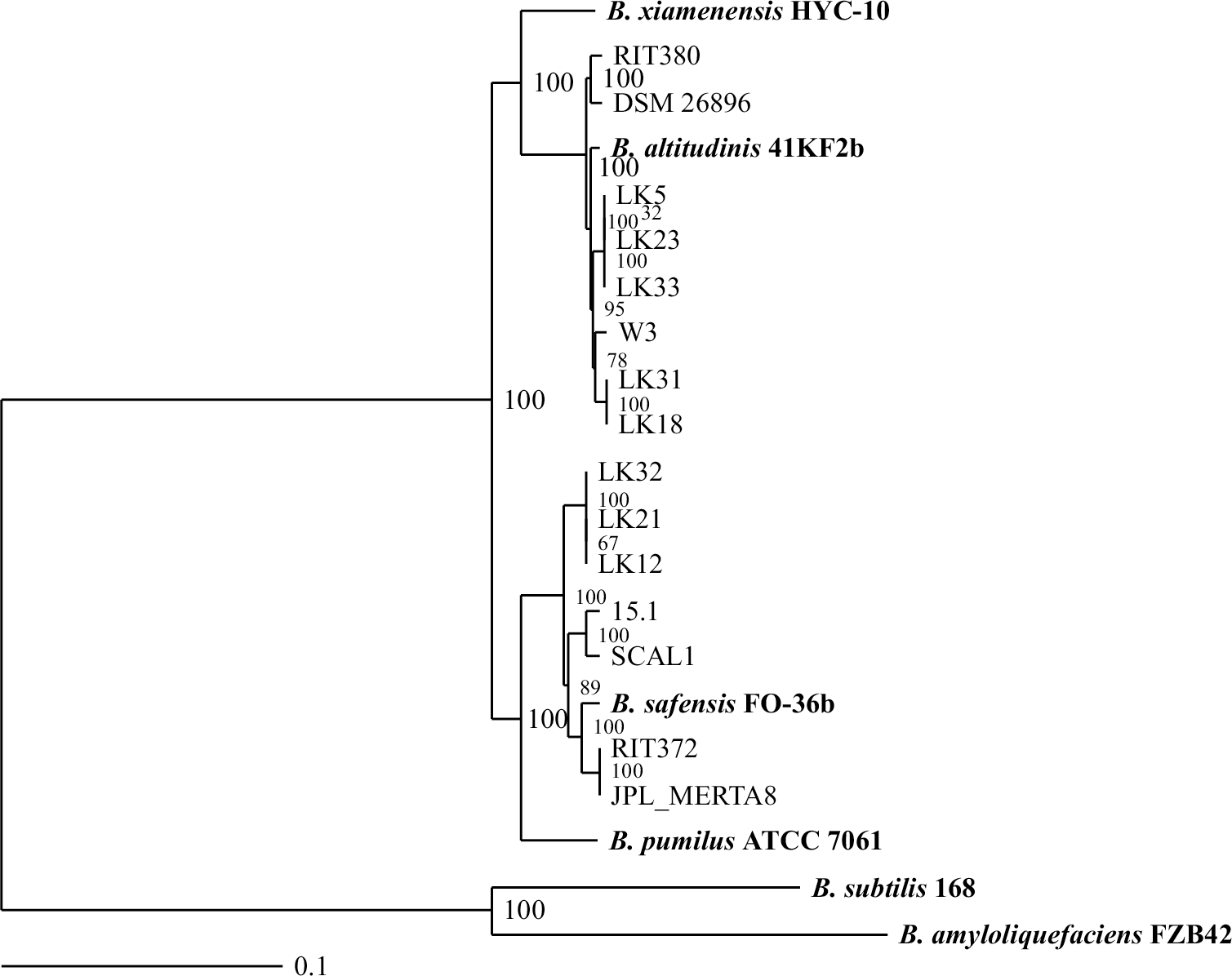

Supplement: S2 Fig — 184 orthologous genes present in all microorganisms under analysis were individually aligned, concatenated and trimmed resulting in a final alignment containing a total of 153853 residues. The evolutionary history of the indicated strains was inferred with RAxML algorithm [24]. Reliability of the inferred tree was tested by bootstrapping with 1000 replicates. Type strains are indicated in bold. (TIF) [file pone.0163098.s002.tif]
